# Prevalence and genetic diversity of *Bartonella* spp. in wild small mammals from South Africa

**DOI:** 10.1128/aem.00842-24

**Published:** 2024-07-26

**Authors:** Tapiwanashe Annamary Mhlanga, Simbarashe Chitanga, Sonja Matthee, Mokgadi Pulane Malatji, Samson Mukaratirwa

**Affiliations:** 1 School of Life Sciences, College of Agriculture, Engineering and Sciences, University of KwaZulu-Natal, Westville Campus, Durban, South Africa; 2 Department of Preclinical Studies, School of Veterinary Medicine, University of Namibia, Windhoek, Namibia; 3 Department of Biomedical Sciences, School of Health Sciences, University of Zambia, Lusaka, Zambia; 4 Department of Conservation Ecology and Entomology, Stellenbosch University, Stellenbosch, South Africa; 5 One Health Centre for Zoonoses and Tropical Veterinary Medicine, Ross University School of Veterinary Medicine, Basseterre, West Indies, Saint Kitts and Nevis; The Pennsylvania State University, University Park, Pennsylvania, USA

**Keywords:** *Bartonella *spp., small mammals, Rodentia, PCR, molecular prevalence, haplotype diversity, genetic diversity, South Africa

## Abstract

*Bartonella* spp. are intracellular bacteria associated with several re-emerging human diseases. Small mammals play a significant role in the maintenance and spread of *Bartonella* spp. Despite the high small mammal biodiversity in South Africa, there is limited epidemiological information regarding *Bartonella* spp. in these mammals. The main aim of this study was to determine the prevalence and genetic diversity of *Bartonella* spp. from wild small mammals from 15 localities in 8 provinces of South Africa. Small mammals (*n* = 183) were trapped in the Eastern Cape, Free State, Gauteng, Limpopo, Mpumalanga, Northern Cape, North West, and Western Cape provinces of South Africa between 2010 and 2018. Heart, kidney, liver, lung, and spleen were harvested for *Bartonella* DNA screening, and prevalence was determined based on the PCR amplification of partial fragments of the 16S–23S rRNA intergenic spacer (ITS) region, *gltA,* and *rpoB* genes. *Bartonella* DNA was detected in *Aethomys chrysophilus*, *Aethomys ineptus*, *Gerbillurus* spp., *Lemniscomys rosalia*, *Mastomys coucha*, *Micaelamys namaquensis*, *Rhabdomys pumilio,* and *Thallomys paedulcus*. An overall prevalence of 16.9% (31/183, 95% CI: 12.2%–23%) was observed. *Bartonella elizabethae*, *Bartonella grahamii,* and *Bartonella tribocorum* were the zoonotic species identified, while the remaining sequences were aligned to uncultured *Bartonella* spp. with unknown zoonotic potential. Phylogenetic analyses confirmed five distinct *Bartonella* lineages (I–V), with lineage IV displaying strong *M. coucha* host specificity. Our results confirm that South African wild small mammals are natural reservoirs of a diverse assemblage of *Bartonella* spp., including some zoonotic species with high genetic diversity, although prevalence was relatively low.

**IMPORTANCE:**

Small mammals play a significant role in the maintenance and spread of zoonotic pathogens such as *Bartonella* spp. Despite the high small mammal biodiversity in southern Africa including South Africa, there is limited epidemiological information regarding *Bartonella* spp. in these mammals across the country. Results from our study showed the liver and spleen had the highest positive cases for *Bartonella* spp. DNA among the tested organs. *Bartonella elizabethae*, *B. grahamii,* and *B. tribocorum* were the three zoonotic species identified and five distinct Bartonella lineages (I–V) were confirmed through phylogenetic analyses. To the best of our knowledge, this study presents the first extensive nuclear diversity investigation of *Bartonella* spp. in South African small mammals in South Africa.

## INTRODUCTION

The genus *Bartonella* consists of facultative, gram-negative, hemotropic α-proteobacteria, which parasitize erythrocytes and endothelial cells of mammalian hosts (1). These bacterial parasites are primarily transmitted by hematophagous arthropod vectors such as lice, mites, and ticks, with fleas considered as the main vectors in small mammal populations (2–6). Over 45 *Bartonella* species isolated from domestic and wild animals have been reported (7), with 15 associated with human infections (8).

*Bartonella* spp. exhibit high specificity to closely related mammalian reservoir hosts displaying a long-lasting bacteremia with no symptoms (3, 9). However, infections in incidental hosts often evoke a wide range of clinical manifestations which include endocarditis (10, 11), myocarditis (12), fever and neurologic disorders (13), meningitis (14), splenomegaly (15), and lymphadenopathy (16). Small mammals play a significant role as reservoirs of *Bartonella* spp. that cause several human medical conditions, including Carrion’s disease, endocarditis, fever, neuroretinitis, and bacteremia, which have been reported globally (17, 18). Furthermore, small mammal-associated Bartonellae are also associated with animal infections which manifest with symptoms that include anorexia, fever, and reproductive disorders in cats (19–21) and endocarditis and hematological disorders such as anemia, neutrophilic leukocytosis, and thrombocytopenia in dogs (22–24).

Research in various countries across the world has demonstrated that small mammals harbor a diverse range of *Bartonella* species, including zoonotic species such as *Bartonella elizabethae* and *Bartonella tribocorum* (25–27), with infection rates as high as 90% in Canada (28, 29), 94% in Japan (30), and 100% in Egypt (31). Despite the abundance of commensal small mammal species in Africa and the high *Bartonella* prevalence rates recorded in other parts of the world, data on the prevalence of *Bartonella* spp. in small mammal reservoirs in most parts of Africa remain limited (32).

Previous studies in South Africa have reported moderate to high prevalence rates (44%–86.7%) of *Bartonella* spp. infection and diversity in wild and peri-domestic small mammals (33–35). However, clinical and epidemiological knowledge regarding *Bartonella* spp. infections in humans, domestic animals, and wildlife is limited (32). Against this background, this study aimed to determine the prevalence and genetic diversity of *Bartonella* spp. in wild small mammal species commonly found in South Africa using multi-locus screening in combination with phylogenetic analyses. Furthermore, this study sought to determine which small mammal organ(s) or tissue(s) are most appropriate for detecting *Bartonella* spp. using conventional PCR.

## MATERIALS AND METHODS

### Study area

**Fig 1:**
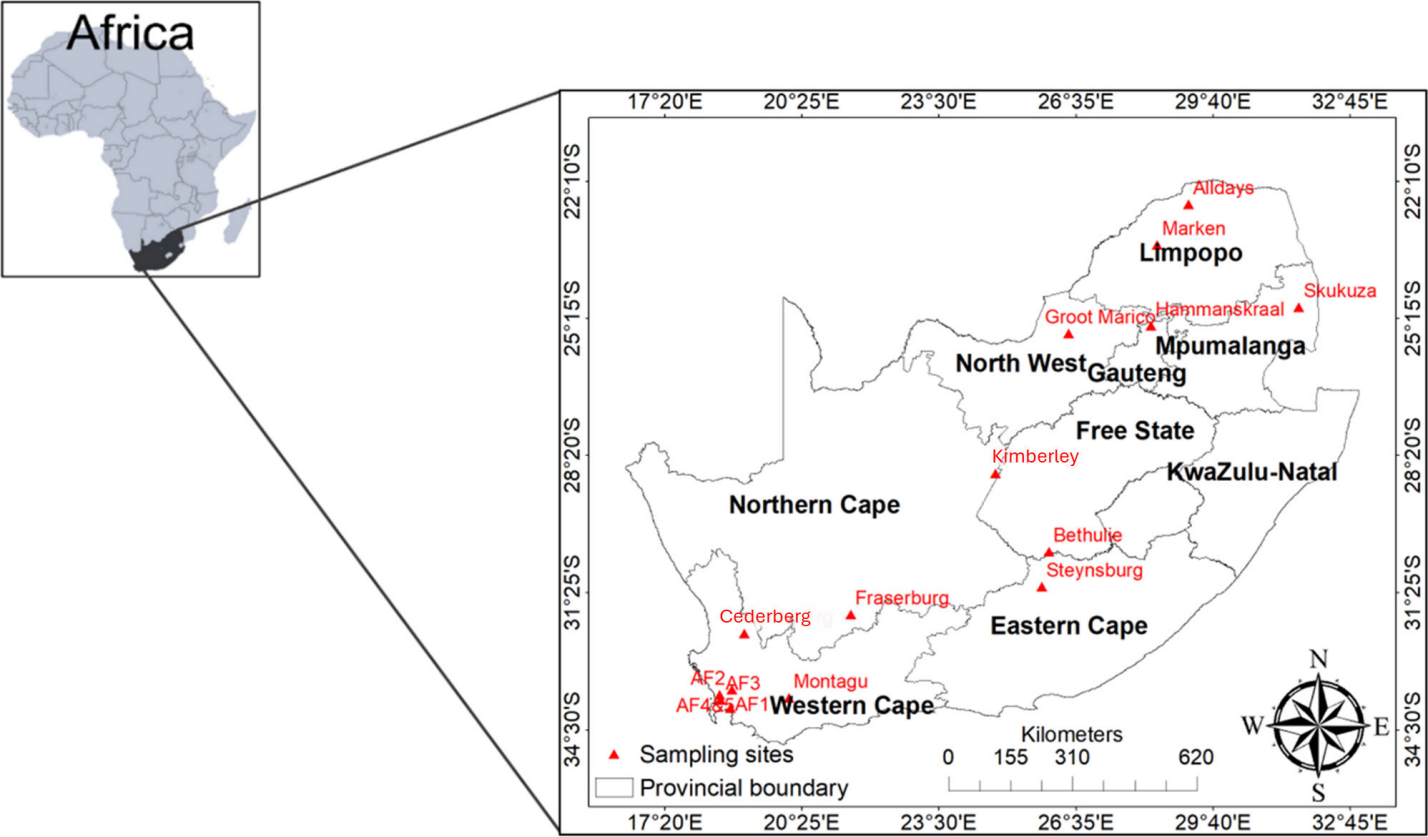
Map of South Africa showing the study localities where small mammals were sampled.

South Africa, the southernmost country on the African continent, is divided into nine administrative provinces (Fig. 1). Weather and climate vary across the provinces, and it becomes hotter and drier from east to west (36). The country consists of nine biomes ranging from Albany thicket, desert, forest, fynbos, grassland, Indian Ocean coastal belt, savanna, succulent, and Nama Karoo (37). The different biomes support a rich diversity of plants that host diverse populations of small mammal species representing several families (38). A total of 15 study localities across 8 provinces of South Africa were selected for this study (Fig. 1). Site selection was based on the abundance and diversity of small mammals in these areas. Representation of different geographic regions was also considered in site selection.

### Collection of study animals

A total of 183 small mammals were trapped from a total of fifteen sampling localities in the Eastern Cape, Free State, Gauteng, Limpopo, Mpumalanga, Northern Cape, North West, and Western Cape between 2010 and 2018 (Fig. 1) (39, 40). Small mammal species screened in this study were *Aethomys chrysophilus, Aethomys ineptus, Gerbillurus paeba, Lemniscomys rosalia, Macroscelides proboscideus, Mastomys coucha, Mastomys natalensis, Micaelamys granti, Micaelamys namaquensis, Rhabdomys pumilio,* and *Thallomys paedulcus*. Information on the number of small mammal species sampled from each sampling locality is shown in Table 1. Small mammal trapping was conducted in agricultural areas and natural pristine vegetation, using Sherman live traps (H.B. Sherman Traps Inc., Tallahassee, FL, USA). Traps were placed 10 m from each other using 100 m line transects (40). The same trapping procedure was maintained at all the localities. Only adult members of each small mammal species were collected to eliminate the confounding factor of age. Small mammals were preliminarily identified morphologically in the field (38, 41) and thereafter molecularly confirmed (mitochondrial cytochrome oxidase subunit I (*COI*) and cytochrome b (*cyt-B*) genes) (39). Small mammals were euthanized using an intraperitoneal injection of 200 mg/kg sodium pentobarbitone. Each small mammal was individually wrapped and frozen in labeled plastic bags in the field. After transportation to the laboratory, the heart, kidney, liver, lung, and spleen were harvested from each small mammal and preserved at −80°C.

**TABLE 1:** Study localities and the number of small mammal species from which organs were harvested for screening of *Bartonella* spp

Study locality	Province	Small mammal species	Sample size (*n*)
Steynsburg	Eastern Cape	Namaqua rock mouse (*Micaelamys namaquensis*)	5
Bethulie	Free State	Namaqua rock mouse (*Micaelamys namaquensis*)	10
Hammanskraal	Gauteng	Namaqua rock mouse (*Micaelamys namaquensis*)	10
		Namaqua rock mouse (*Mastomys coucha*)	2
Alldays	Limpopo	Red rock rat (*Aethomys chrysophilus*)	1
		Bushveld gerbil (*Gerbillurus leucogaster*)	1
		Natal multimammate mouse (*Micaelamys namaquensis*)	8
Marken	Limpopo	Tete veld rat (*Aethomys ineptus*)	10
		Namaqua rock mouse (*Micaelamys namaquensis*)	8
		Acacia rat (*Thallomys paedulcus*)	1
Skukuza (Kruger National Park)	Mpumalanga	Tete veld rat (*Aethomys ineptus*)	10
		Single-striped grass mouse (*Lemniscomys rosalia*)	4
		Natal multimammate mouse (*Mastomys natalensis*)	2
Kimberley	Northern Cape	Namaqua rock mouse (*Micaelamys namaquensis*)	10
Fraserburg	Northern Cape	Namaqua rock mouse (*Micaelamys namaquensis*)	10
Groot Marico	North West	Tete veld rat (*Aethomys ineptus*)	3
		Single-striped grass mouse (*Lemniscomys rosalia*)	3
		Southern multimammate mouse (*Mastomys coucha*)	13
Agricultural Fragment 1 (Stellenbosch NU)	Western Cape	Four-striped mouse (*Rhabdomys pumilio*)	10
Agricultural Fragment 2 (Cape Farms, Cape Town)	Western cape	Four-striped mouse (*Rhabdomys pumilio*)	10
Agricultural Fragment 3 (Cape Farms, Durbanville)	Western Cape	Four-striped mouse (*Rhabdomys pumilio*)	10
Agricultural Fragment 4 & 5 (Drakenstein)	Western Cape	Four-striped mouse (*Rhabdomys pumilio*)	8
Cederberg	Western Cape	Hairy-footed gerbil (*Gerbillurus paeba*)	2
		Namaqua rock mouse (*Micaelamys namaquensis*)	10
		Round-eared elephant shrew (*Macroscelides proboscideus*)	4
		Four-striped mouse (*Rhabdomys pumilio*)	4
Montagu	Western Cape	Hairy-footed gerbil (*Gerbillurus paeba*)	3
		Four-striped mouse (*Rhabdomys pumilio*)	10
		Round-eared elephant shrew (*Macroscelides proboscideus*)	1
Total			183

### DNA extraction and purification

Genomic DNA was extracted using the phenol-chloroform DNA extraction method (42) from the heart, kidney, liver, lung, and spleen of sampled small mammals (previously preserved at −80°C). Molecular-grade nuclease-free water was used as a blank control during extraction to confirm the purity of the extraction reagents. The quality of the extracted DNA was assessed using agarose gel electrophoresis, where no smearing indicated good-quality DNA. The NanoDrop ND-1000 Spectrophotometer (Thermo Fisher Scientific, USA) was used to measure DNA quantity, and samples with yield below 20 ng/µL were re-extracted. Samples with a DNA concentration >100 ng/µL were diluted with elution buffer to a DNA concentration of 50–100 ng/μL for each sample to reduce the risk of PCR inhibition by excessive DNA. The 260/280 absorbance ratio that denotes DNA purity was also used to check DNA quality, and samples that did not fall within the 1.8–2.0 range were re-extracted. Molecular-grade nuclease-free water was used as a negative control between measurements. DNA extracts were stored at −80°C in 150 µL TE buffer until further processing.

### Identification of small mammal species

Accurate species identification using morphological characters among African small mammals is often challenging due to the existence of cryptic and sibling species complexes, which often do not possess distinguishable external morphological features (38, 41). Molecular identification was used to supplement and confirm *in situ* morphological identification of small mammals in this study. The mitochondrial cytochrome oxidase subunit I (*COI*) gene was amplified for all small mammals using primer set LCO1490 (5′-GGTCAACAAATCATAAAGATATTGG-3′) and HCO2198 (5′-TAAACTTCAGGGTGACCAAAAAATCA-3′) (43) following thermocycling conditions: 5 min of initial denaturation at 94°C, followed by 35 cycles of denaturation for 1 min at 94°C, 1 min of annealing at 48°C and 1 min of extension at 72°C, and a final extension of 10 min at 72°C. This PCR was also used to evaluate PCR inhibition and establish the presence of amplifiable DNA in the extracted samples. To confirm species authenticity, the cytochrome *b* gene regions were also amplified and sequenced using the primer pair L14816 (5′-CCATCCACCATCTCAGCATGATGAAA-3′) and H15173 (5′-CCCCTCAGCATGATATTTGTCCTCA-3′) (44) following thermocycling conditions: 5 min of initial denaturation at 95°C, followed by 35 cycles of denaturation at 94°C for 30 s, annealing at 50°C for 45 s and extension at 72°C for 1 min, and final extension of 7 min at 72°C. All amplifications were performed in a 25 µL volume containing 4 µL template DNA, 2 µL of each primer (10 mM), 12.5 µL Quick-Load Taq 2X Master Mix (New England BioLabs), and 4.5 µL ultra-pure molecular-grade water. For the negative controls, the DNA template was replaced with ultra-pure molecular-grade water. Fragments were separated on a 1.5% agarose gel stained with ethidium bromide and viewed under UV light compared to 100 bp and 1 kb DNA molecular weight markers (New England BioLabs). Successful amplicons showed bands on 465 bp (*COI*) and 357 bp (*cyt-B*) and were subsequently sent to Inqaba Biotechnological Industries (Pretoria, South Africa) for purification and unidirectional Sanger sequencing. To confirm the taxonomic identity of the small mammals, obtained *COI* and *cyt-B* sequences were compared against data in the GenBank database using the nucleotide BLAST (BLASTN) function.

### PCR detection of *Bartonella* spp. DNA

Conventional PCR was used to detect the presence of *Bartonella* spp. DNA using a multi-locus sequence analysis (MLSA) approach. The spleen DNA extracts were used for the initial screening of *Bartonella* spp. The heart, kidney, liver, and lung samples were subsequently screened for *Bartonella* spp. DNA to determine the dissemination/presence of the bacteria in the different organs of each infected individual. However, for some of the selected small mammals, not all organs were available for screening. Amplification protocols using *Bartonella* primers only differed in annealing temperatures. Primers 321s (5′-AGATGATGATCCCAAGCCTTCTGG-3′) and 983as (5′-TGTTCTYACAACAATGATGATG-3′) developed by Maggi and Breitschwerdt (45) were used to amplify the *Bartonella* 16S-23S rRNA ITS region (453–717 bp) using the following reaction conditions: 5 min of initial denaturation at 95°C, followed by 45 cycles of denaturation at 94°C for 45 s, annealing at 54°C for 45 s, and extension at 72°C for 45 s, and final extension of 72°C for 10 min. Primers BhCS.781p (5′-GGGGACCAGCTCATGGTGG-3′) and BhCS.1137n (5′-AATGCAAAAAGAACAGTAAACA-3′) by Norman et al. (46) were used to amplify a 379 bp *Bartonella gltA* region using the reaction conditions as described above, with an annealing temperature of 52°C. PCR amplification of *Bartonella rpoB* 825 bp fragments was performed with primers 1400F (5′-CGCATTGGCTTACTTCGTATG-3′) and 2300R (5′-GTAGACTGATTAGAACGCTG-3′) (47) with annealing temperature 52°C. All PCRs were performed in a 25 µL total reaction volume. Each reaction consisted of 12.5 µL Quick-Load Taq 2X Master Mix (New England BioLabs), 2 µL of 10 mM of each primer (forward and reverse), 4.5 µL ultra-pure molecular grade water, and 4 µL template DNA. For the negative controls, the DNA template was replaced with ultra-pure molecular-grade water. The presence of amplicons of the expected size was established by running 2 µL of PCR product on 1.5% agarose gel stained with ethidium bromide and visualized under UV light compared to 100 bp and 1 kb DNA molecular weight markers (New England BioLabs). Positive PCR products were sent for purification and unidirectional sequencing to Inqaba Biotechnological Industries (Pretoria, South Africa).

### Sequence editing and phylogenetic analysis

Sequences were assembled and edited using BioEdit Sequence Alignment Editor version 7.2.5 (48), and compared to sequences from the GenBank database using the NCBI Blast program. Edited sequences were aligned with homolog sequences from the GenBank database using the MUSCLE alignment tool in MEGA X (49) jModeltest (50) was used to select the best model test for nucleotide substitution and the following models were selected; HKY + G + I model based on a 908 bp alignment of the *Bartonella rpoB* gene, the T92 +G + I model based on an 877 bp alignment of the *Bartonella gltA* gene, T92 +G model based on a 1,242 bp alignment of the *Bartonella* 16 S-23S rRNA ITS region, and the HKY + G + I model based on a 2,964 bp alignment of concatenated *Bartonella* 16S-23S rRNA ITS region, *gltA* and *rpoB* genes. Maximum likelihood trees were generated using MEGA X, with nodal support estimated using 1,000 bootstrap pseudo-replicates. Bayesian inference analysis was conducted using MrBayes 3.1.2 (51) using four Markov chains, with 10^6^ generations, a sampling frequency of 500, a diagnostic frequency of 5,000, and a burn-in of 25%. Analysis was run until the split frequencies standard deviation was less than 0.01, the potential scale reduction factor (PSRF) was close to 1.0 for all parameters, and the effective sample size (ESS) was over 100.

### Haplotype (genetic diversity) analysis

DnaSP version 6.12.03 (52) software was used to calculate the genetic diversities and evaluate sequence polymorphism. The number of variable sites (VS), number of haplotypes (h), haplotype diversity (Hd), nucleotide diversity (π), number of nucleotide differences (K), and the standard deviation (SD) were calculated. Haplotype networks were generated using the Population Analysis with Reticulate Trees (PopArt) software version 1.7 using the TCS and median-joining networks (53, 54).

### Statistical analysis

*Bartonella* spp. infection prevalence rates were calculated using the formula adapted from Thrusfield (55), then categorized and summarized according to sampling locality, province, and tested small mammal organ. The 95% confidence intervals (CI) were calculated, and a Chi-square test was used to test for significant differences in *Bartonella* spp. prevalence in the different sampling localities, provinces, and tested small mammal organs, where *P*-values ≤ 0.05 were considered significant. All statistical analyses were conducted using IBM SPSS software version 22.0 (SPSS, Inc., Chicago, IL, USA).

## RESULTS

### Detection of *Bartonella* spp. in small mammals

*Bartonella* spp. DNA was successfully amplified in 31/183 (16.9%, 95% CI: 12.2%–23%) screened small mammals (Table 2). Of the 31 small mammals positive for *Bartonella* spp., 9/31 (29%) were identified as *M. coucha*, 6/31 (19.4%) as *A. ineptus*, 1/31 (3.2%) as *A. chrysophilus*, 4/31 (12.9%) as *Gerbillurus* spp., 1/31 (3.2%) as *L. rosalia*, 5/31 (16.1%) as *M. namaquensis*, 4/31 (12.9%) as *R. pumilio,* and 1/31 (3.2%) as *T. paedulcus* (Table 2).

**TABLE 2:** Prevalence of *Bartonella* spp. from small mammals sampled in various localities of eight provinces of South Africa

Study locality	Province	Small mammal species	Sample size (n)	No. positive	Prevalence (%)	95%CI
Steynsburg	Eastern Cape	*Micaelamys namaquensis*	5	0	0	0.0–43.5
Bethulie	Free State	*Micaelamys namaquensis*	10	0	0	0.0–27.8
Hammanskraal	Gauteng	*Micaelamys namaquensis*	10	0	8.33	1.5–35.4
		*Mastomys coucha*	2	1		
Alldays	Limpopo	*Aethomys chrysophilus*	1	1	40	16.8–68.7
		*Gerbillurus leucogaster*	1	1		
		*Micaelamys namaquensis*	8	2		
Marken	Limpopo	*Aethomys ineptus*	10	3	21.1	8.5–43.3
		*Micaelamys namaquensis*	8	0		
		*Thallomys paedulcus*	1	1		
Skukuza (Kruger National Park)	Mpumalanga	*Aethomys ineptus*	10	2	12.5	3.5–36.0
		*Lemniscomys rosalia*	4	0		
		*Mastomys natalensis*	2	0		
Kimberley	Northern Cape	*Micaelamys namaquensis*	10	1	10	1.8–40.4
Fraserburg	Northern Cape	*Micaelamys namaquensis*	10	1	10	1.8–40.4
Groot Marico	North West	*Aethomys ineptus*	3	1	52.6	31.7–72.7
		*Lemniscomys rosalia*	3	1		
		*Mastomys coucha*	13	8		
Agricultural Fragment 1 (Stellenbosch NU)	Western Cape	*Rhabdomys pumilio*	10	2	20	5.7–51.0
Agricultural Fragment 2 (Cape Farms, Cape Town)	Western Cape	*Rhabdomys pumilio*	10	1	10	1.8–40.4
Agricultural Fragment 3 (Cape Farms, Durbanville)	Western Cape	*Rhabdomys pumilio*	10	0	0	0.0–27.8
Agricultural Fragment 4&5 (Drakenstein)	Western Cape	*Rhabdomys pumilio*	8	0	0	0.0–32.4
Cederberg	Western Cape	*Gerbillurus paeba*	2	1	10	2.8–30.1
		*Micaelamys namaquensis*	10	1		
		*Macroscelides proboscideus*	4	0		
		*Rhabdomys pumilio*	4	0		
Montagu	Western Cape	*Gerbillurus paeba*	3	2	21.4	7.6–47.6
		*Rhabdomys pumilio*	10	1		
		*Macroscelides proboscideus*	1	0		
Total			183	31	16.9	12.2–23.0

### Detection and prevalence of *Bartonella* spp. in organs of small mammals

**Fig 2:**
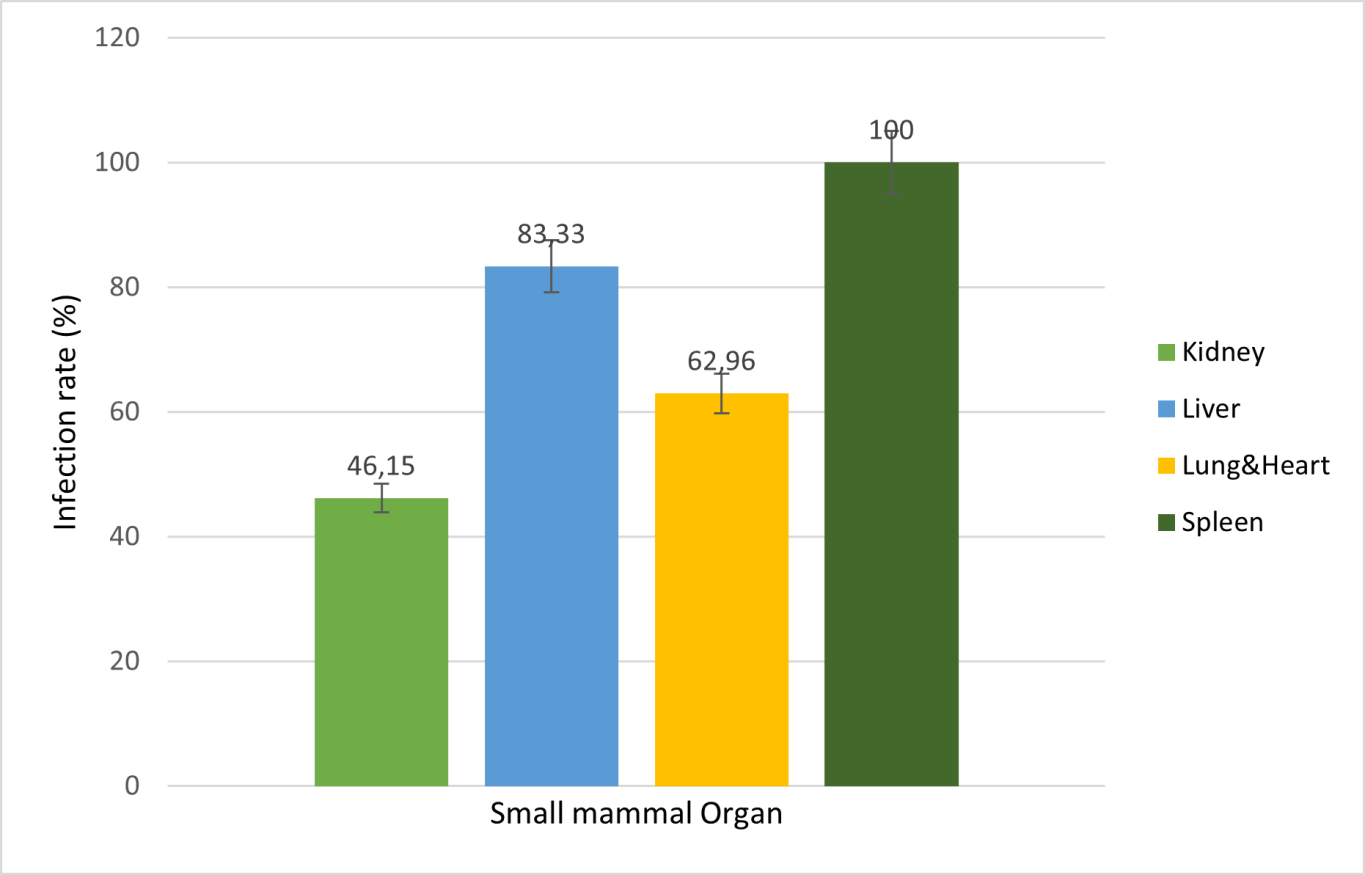
Prevalence of *Bartonella* spp. infection of small mammals by organ screened. The comparison of prevalence between the different small mammal organs only concerns the 31 *Bartonella*-positive small mammals.

Of the organs screened, the spleen had the highest number of positive cases (31/31, 100%), followed by the liver (20/24, 83.3%), heart and lungs (17/27, 63.0%), and the kidney had the lowest infection rate (12/26, 46.2%) (Fig. 2). Infection rates significantly differed among different organs (*X*^2^ = 7.463, *P*-value 0.024).

### Prevalence of *Bartonella* spp. from small mammals between sampling localities

*Bartonella* spp. DNA was detected in small mammals from 11 of the 15 study localities, and infection rates differed significantly across the sampling sites (*x*^2^ = 29.896, *P*-value = 0.01) and all eight provinces (Pearson’s *x*^2^ = 24.234, *P*-value < 0.001). The highest prevalence of 52.6% (10/19, 95% CI: 31.7%–72.7%) was observed in small mammals from Groot Marico (Table 2). All small mammals sampled from Agricultural Fragments 3, 4, and 5, Steynsburg and Bethulie tested negative for *Bartonella* spp. infection.

### *Bartonella* spp. identification based on BLASTn analysis

A total of 39 *rpoB*, 14 *gltA,* and 8 ITS sequences were generated and analyzed. Despite several attempts, the 16S-23S rRNA ITS region and *gltA* loci could not be consistently amplified for all *rpoB*-positive samples due to variable PCR assay performance. Nucleotide BLASTn of *rpoB* sequences showed similarity ranging from 93% to 100% with *Bartonella* spp. isolates from small mammals from the Democratic Republic of Congo, Ethiopia, South Africa, and Tanzania. Notably, isolate GMMN6 from our study had a 99% similarity with pathogenic *B. elizabethae* (LR134527.1), and isolate OBGP3 from a gerbil from Montagu had a 95% similarity with *B. tribocorum* (JF766251.1).

BLASTn of *gltA* sequences showed a similarity index of between 97% and 100% with *Bartonella* spp. *Bartonella* isolates from two *M. coucha*, GMMN1 and GMMN4, were 100% identical to previously described *Bartonella* sp. AN-nh3 (AJ583114.1) from small mammals in South Africa. Isolate GMMN6 from Groot Marico had a 100% match with pathogenic *B. elizabethae* (GU056192.1) previously isolated from ectoparasites from stray cats (*Felis catus*) in Taiwan. Sequence MOMN6 had a 98% match with *B. tribocorum* (KT327031.1) isolated from a Libyan jird. Two sequences from *M. coucha* from Groot Marico (GMMN1 and GMMN4) had a similarity of 96% to *B. grahamii* as4aup (CP001562.1) previously isolated from a wood mouse (*Apodemus sylvaticus*) captured in Sweden.

Percentage similarity of *Bartonella* spp. isolates detected based on the 16S-23S rRNA ITS region ranged from 87% to 100%. Isolate GMMN7 from *M. coucha* from Groot Marico was 100% similar to *Bartonella* sp. An27ug (JX428757.1) isolated from *Arvicanthis niloticus* in Uganda. Compared to the other two loci used, the ITS had a much lower range of similarity values with sequences from GenBank. The majority of the closest related isolates, according to the BLASTn analysis for all the three loci used in this study, were small mammal-associated Bartonellae, mostly from sub-Saharan Africa.

### Phylogenetic analyses of *Bartonella* spp. from eight provinces of South Africa

**Fig 3:**
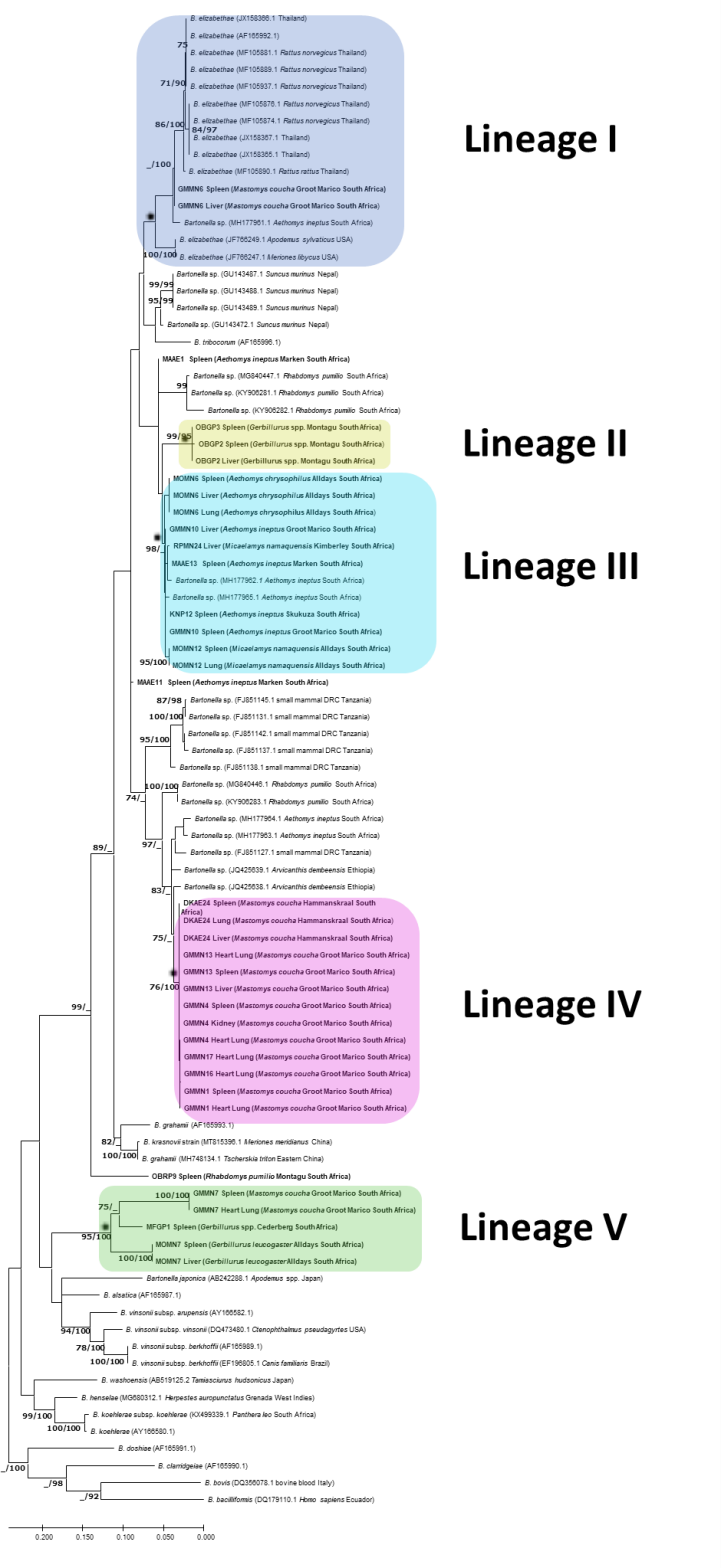
Phylogenetic tree inferred by Maximum Likelihood (ML) based on a 908 bp alignment of the *Bartonella rpoB* gene. Numbers at nodes represent ML bootstrap support values with 1,000 repetitions and Bayesian posterior probabilities (BPP) greater than 70% (ML/BPP). Sequences from this study are shown in bold.

Phylogenetic analysis identified *Bartonella rpoB* sequences from this study clustered within five distinct lineages I–V (Fig. 3). Lineage I contained sample GMMN6 (*M. coucha*) which clustered with *B. elizabethae* isolates from small mammals from the United States and Thailand. The lineage also included a *Bartonella* isolate from *A. ineptus* previously described in South Africa.

Lineage II consisted of isolates from two *Gerbillurus* species from Montagu, and this lineage appeared to be *Gerbillurus*-specific. Lineage III contained isolates from *A. ineptus*, *A. chrysophilus,* and *M. namaquensis* from Groot Marico, Marken, Alldays, and Skukuza (Kruger National Park).

Lineage IV, the dominant lineage in *M. coucha*, contained isolates from Groot Marico and one sample from Hammanskraal, and this lineage appeared to be *Mastomys*-specific. Lineage V consisted of samples from *Gerbillurus* species from Cederberg and Alldays and one isolate from *M. coucha* from Groot Marico.

**Fig 4:**
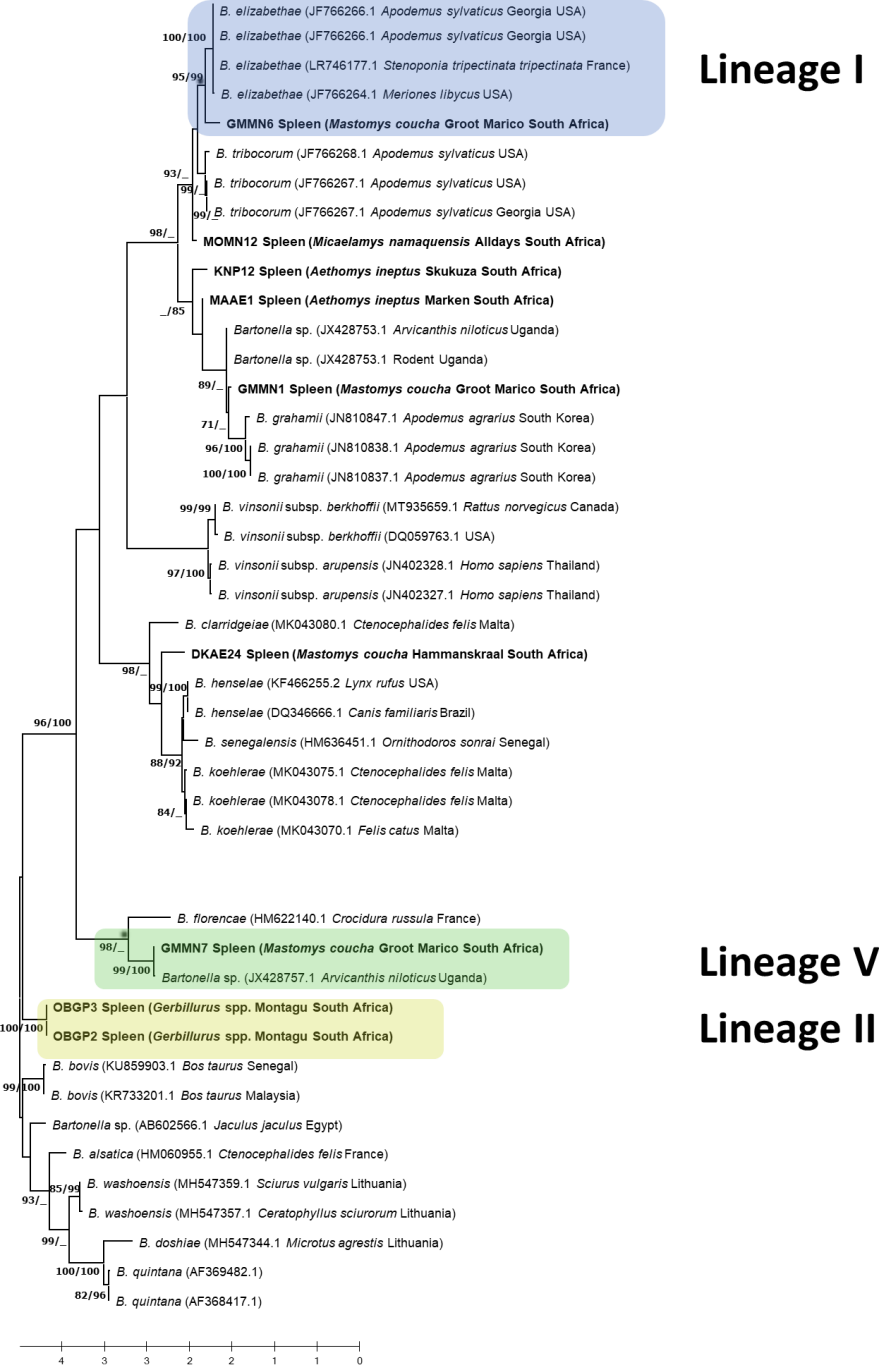
Phylogenetic tree inferred by Maximum Likelihood (ML) based on a 1,242 bp alignment of the *Bartonella* 16S-23S rRNA ITS region. Numbers at nodes represent ML bootstrap support values with 1,000 repetitions and Bayesian posterior probabilities (BPP) greater than 70% (ML/BPP). Sequences from this study are shown in bold.

**Fig 5:**
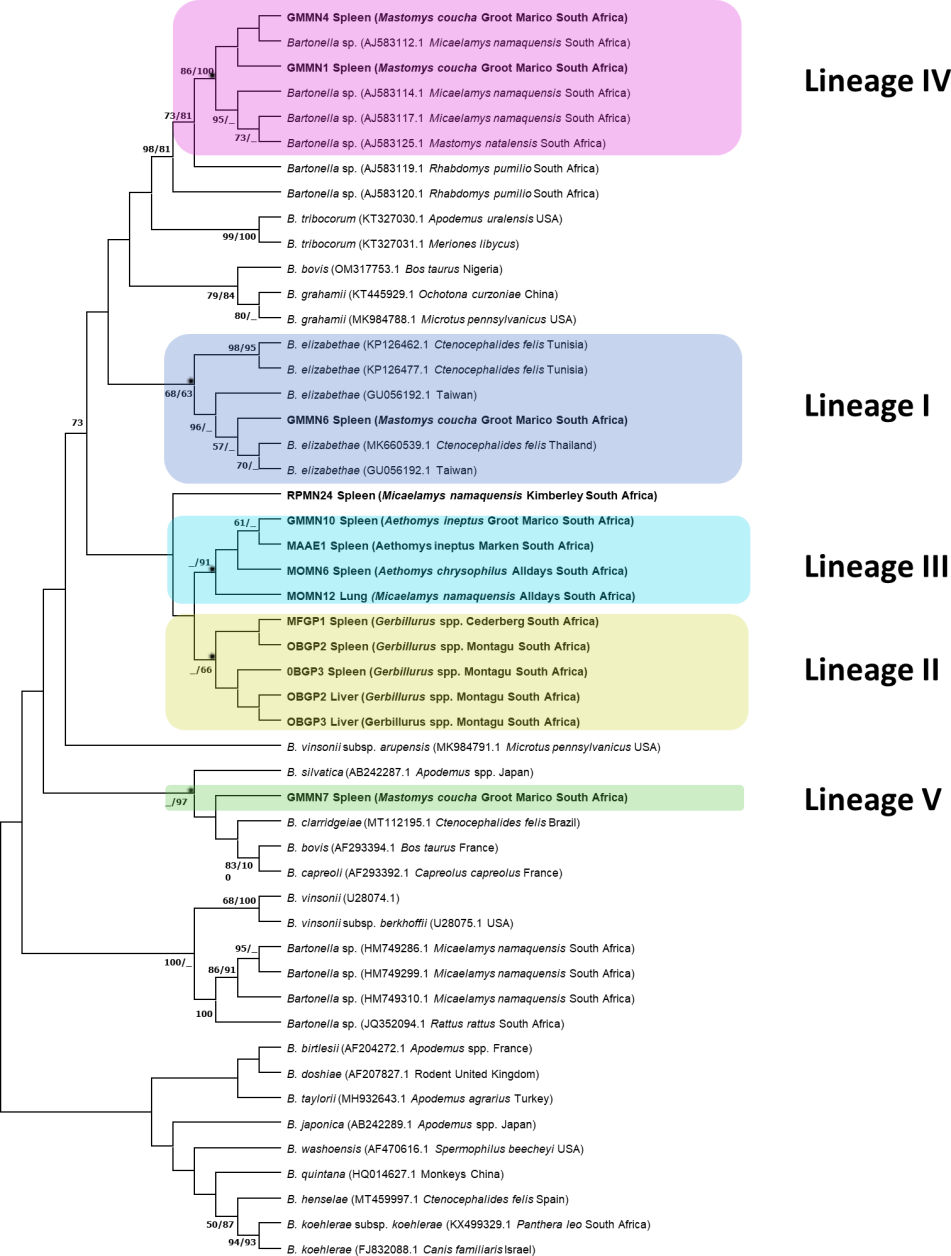
Phylogenetic tree inferred by Maximum Likelihood (ML) based on an 877 bp alignment of the *Bartonella gltA* gene. Numbers at nodes represent ML bootstrap support values with 1,000 repetitions and Bayesian posterior probabilities (BPP) greater than 70% (ML/BPP). Sequences from this study are shown in bold.

Lineages II and IV showed a strong *Gerbillurus* and *Mastomys* host association, respectively. Despite variable assay performance, the clustering of the five lineages was consistent with the 16S-23S rRNA ITS region (Fig. 4), *gltA* (Fig. 5), and concatenated *gltA*, ITS, and *rpoB* (Fig. S1) gene phylogenies. For all three gene loci used in this study, all five lineages were in well-supported clades based on Maximum Likelihood bootstrap values over 70% and Bayesian posterior probabilities greater than 90%.

Lineage II (*Gerbillurus*-specific clade) and Lineage III (*Aethomys* and *Micaelamys*-specific clade) were more closely related and clustered together, whereas Lineage IV (*Mastomys*-specific clade) and Lineage V (*Mastomys* and *Gerbillurus*-specific clade) clustered together. Lineage I, which consisted of *B. elizabethae* isolates and a *Bartonella* spp. isolate from *M. coucha* from Groot Marico, was distinct and did not cluster with any of the other lineages from this study.

It was not possible to generate data for all five lineages across the three loci due to varied PCR assay performance, hence the fewer samples for the *gltA* and 16S-23S rRNA ITS phylogeny analysis in comparison to the *rpoB* locus.

### Genetic diversity and haplotype analysis of *Bartonella* spp

**Fig 6:**
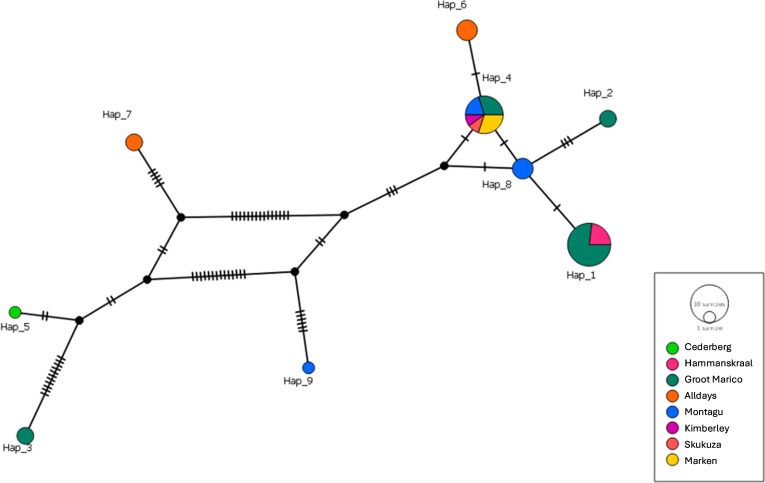
Haplotype analysis of *Bartonella rpoB* isolates from wild small mammals collected from eight provinces of South Africa. The haplotype network was constructed in PopArt using the TCS network.

Analysis of 21 *rpoB* sequences yielded a total of 9 haplotypes (Fig. 6), with an overall haplotype diversity of 0.824 (Table 3). Haplotypes 1, 2, 4, 6, and 8 were much more closely related and distinct from haplotypes 3, 5, 7, and 9, which had comparatively more mutational events between them. The genetic diversity of isolates from this study, inferred by analysis of *rpoB* sequences, differed significantly between the study sites (*x*^2^ = 72.9, *P*-value < 0.001). Groot Marico had the most haplotypes detected, with a moderately high haplotype diversity of 0.632. The highest haplotype diversity was observed Alldays (0.761) in Limpopo province, with three haplotypes detected (Table S2). Of the nine distinct haplotypes (Fig. 6), haplotype 4 was the most representative and consisted of sequences from Groot Marico, Montagu, Marken, Kimberley, and Skukuza (Kruger National Park).

**TABLE 3:** Genetic diversity of *Bartonella* spp. sequences detected in small mammals in this study based on the 16S-23S rRNA ITS region, *rpoB,* and *gltA* genes^*a*^

Species	Gene/region	(bp)	N	VS	h	Hd (mean ± SD)	π (mean ± SD)	K
*Bartonella* spp.	*rpoB*	602	21	46	9	0.824 ± 0.060	0.02217 ± 0,00673	9.357
	*gltA*	293	12	14	5	0.788 ± 0.008	0.01917 ± 0.00486	3.758
	16S-23S rRNA ITS	955	9	45	8	0.972 ± 0.064	0.12739 ± 0.01832	18.472

^*a*^ Only *Bartonella* spp. isolates detected in this study were used in the analysis**;** N, number of sequences analyzed; VS, number of variable sites; h, number of haplotypes; Hd, diversity of haplotypes; SD, standard deviation; π, nucleotide diversity (per site); K, number of nucleotide differences.

*Bartonella* spp. from different organs of the same infected small mammal host all clustered in similar haplotype groups. The *Bartonella rpoB* sequences in haplotype 4 were from *A. ineptus*, *M. coucha,* and *M. namaquensis*. Haplotypes 6 and 8 were separated from Haplotype 4 by a single mutational event. Haplotype 1 was the most frequently detected, with sequences mostly from Groot Marico and a few from Hammanskraal, but they were all from *M. coucha*.

**Fig 7:**
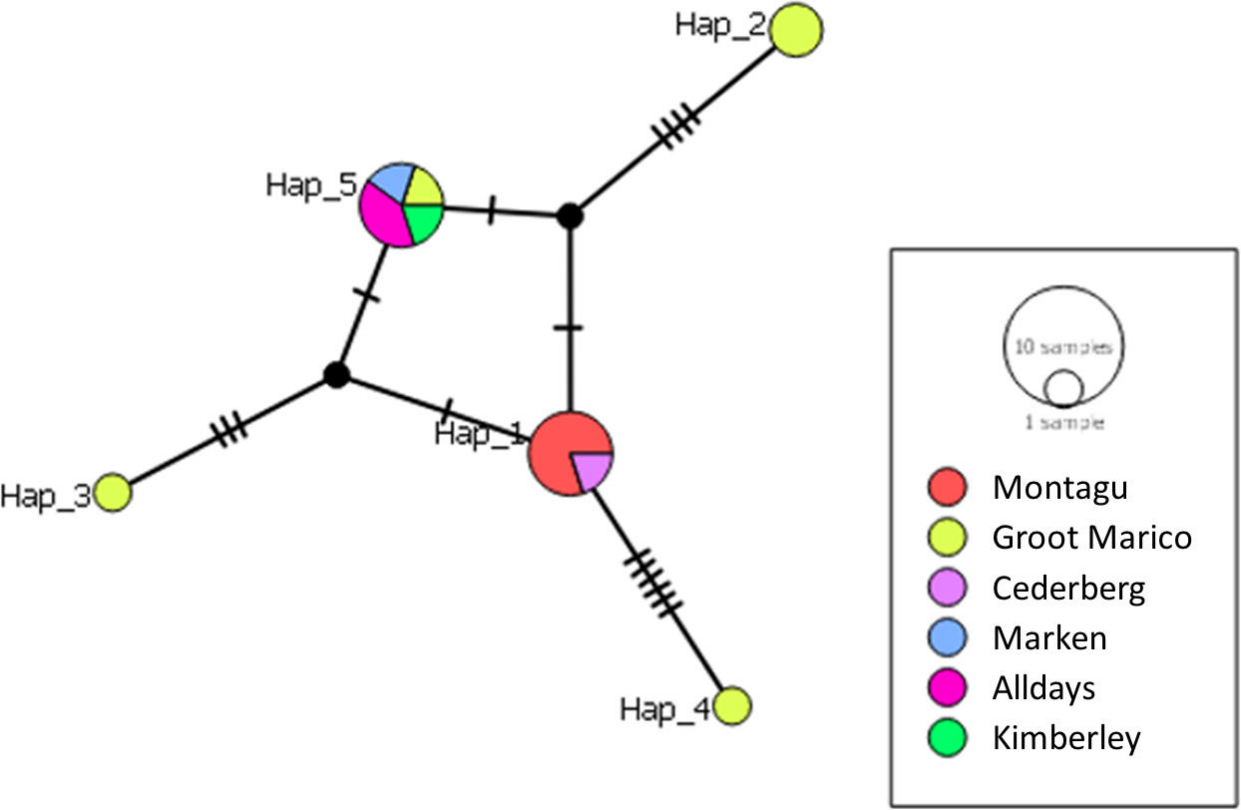
Haplotype analysis of *Bartonella gltA* isolates from wild small mammals from this study. The haplotype network was constructed in PopArt using the Median Joining network method (Epsilon = 0).

Twelve *gltA* sequences yielded five haplotypes, with an overall haplotype diversity of 0.788 (Table 3). The haplotype diversity of 12 *gltA* sequences differed significantly across study sites (*x*^2^ = 16.867, *P*-value < 0.03). Groot Marico had the most haplotypes detected, with a high haplotype diversity of 0.9 and nucleotide diversity of 0.03590 while Alldays and Montagu had one haplotype each (Table S3). A median-joining haplotype network revealed five distinct haplotypes (Fig. 7). Haplotypes 2, 3, and 4 consisted of *Bartonella* spp. isolates from *M. coucha* small mammals from Groot Marico. Haplotype 5 was the most representative and consisted of isolates from Groot Marico, Montagu, Marken, Kimberley, and Skukuza (Kruger National Park). Haplotypes 1 and 5 were the most common haplotypes, with five isolates each. The clustering of the *gltA* haplotypes was consistent with that observed with the *rpoB* haplotype analysis.

**Fig 8:**
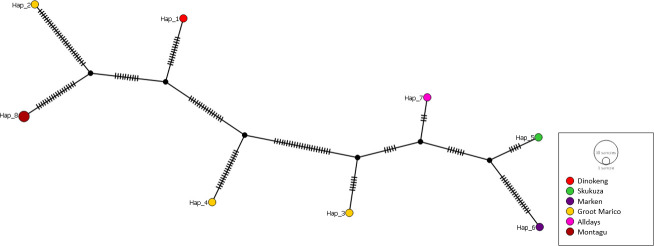
Haplotype analysis of *Bartonella* 16S-23S rRNA ITS region isolates from small mammals from this study. The haplotype network was constructed in PopArt using the TCS network.

From the nine 16S-23S rRNA ITS sequences analyzed, a total of eight haplotypes (Fig. 8) were generated with an overall haplotype diversity of 0.972 (Table 3). However, the haplotype diversity was not significantly different between the study sites (*x*^2^ = 5, *P*-value = 0.17). The distribution of the isolates across the haplotypes was consistent with that of *rpoB* and *gltA* haplotype analyses.

The clustering of haplotypes for all three gene loci correlated with the structuring of the phylogenetic trees. A degree of host specificity was observed in the haplotype networks, with haplotypes grouping mostly according to host species. Haplotypes were not organ or locality-specific, as observed in the phylogenetic trees.

## DISCUSSION

*Bartonella* spp. infection was detected in eight small mammal species, namely *A. chrysophilus*, *A. ineptus*, *Gerbillurus* spp. *L. rosalia*, *M. coucha*, *M. namaquensis*, *R. pumilio,* and *T. paedulcus*, and to the best of our knowledge, this is the first report of *Bartonella* spp. in *T. paedulcus* in South Africa. An overall infection rate of 16.9% (31/183) was observed, and the spleen and liver were the most sensitive tissues for the detection of *Bartonella* spp. using conventional PCR. The observed overall prevalence in this study is in range with previously detected prevalence in small mammals from the Democratic Republic of Congo, Ethiopia, Japan, and South Africa (30, 56–58), but was much lower than that previously reported in South Africa (44%– 86.7%) (33–35, 57).

Previous studies have reported strong associations between some small mammal species and *Bartonella* infection rates in wild rodent populations (35). Among the small mammal species screened in this study, the highest prevalence was observed in *M. coucha* (9/31, 29%)*, A. ineptus* (6/31, 19.4%), and *M. namaquensis* (5/31, 16.1%). The consistently high prevalence rates observed in *M. coucha* across various localities in this study point to predisposing host characteristics in *M. coucha,* such as high reproductive rates that result in naïve offspring getting infected (59). The high infection rate could also be due to the inability of *M. coucha* to resolve *Bartonella* infection after a certain period, unlike other small mammals like *Myodes glareolus,* where quick resolution has been reported, leading to longer persistence of infection in this small mammal species (59). The high *Bartonella* infection prevalence in the commensal rodent species, *M. coucha*, emphasizes the importance of this species in the potential spread of zoonotic *Bartonella* spp. in South Africa, particularly in areas where humans live in proximity to the commensal rodent. This rodent species may be used as a sentinel species in *Bartonella* surveillance programs (60).

*Bartonella* infection in *Lemniscomys* spp. has been previously reported by Halliday et al. (61) in rural Kenya. Previous studies conducted in South Africa have demonstrated that indigenous small mammals are carriers of a diverse range of *Bartonella* spp. strains, with infection rates of 15% in *R. pumilio* (58), 44% in *M. namaquensis* (34), and a high prevalence of 86.7% in *A. ineptus* (35). Like in previous studies, the data in the present study also indicated significant differences between *Bartonella* spp. infection at different sampling localities (56, 57).

A level of *Bartonella* host specificity was observed, with certain lineages containing *Bartonella* spp. from specific small mammal species only. Lineage IV consisted of *M. coucha* isolates from this study only and appeared to be *Mastomys*-specific. However, information on GenBank sequences and analysis also showed that genetically identical *Bartonella* spp. isolates were previously reported to infect different small mammal species. This imperfect host specificity of *Bartonella* spp. in small mammals has been observed and reported in the United Kingdom where one *Bartonella* species infected five different small mammal species (62), and in laboratory-reared cotton rats (*Sigmodon hispidus*) which were successfully experimentally infected with three different *Bartonella* spp. (63). In a study conducted in Sweden, a *Bartonella* spp. isolate identical to *B. grahamii* was isolated from two rodent species, *Apodemus flavicollis* and *Mus musculus* (64).

Based on the *gltA*, *rpoB,* and ITS gene sequence analyses, *Bartonella* spp. isolates detected in the different tissues of each small mammal species were consistent. This agrees with the findings from Yu et al. (65) where *Bartonella* spp. detected in different tissues (liver, kidney, and spleen) of tested small mammals were found to be consistent. There was an observed difference in the infection rate between organs, with the spleen and liver showing a higher infection rate as compared to the kidney. The observed differences in the infection rate were significantly different and agreed with the findings reported in small mammals from Nepal by Gundi et al. (26). Furthermore, Deng et al. (66) confirmed the accretion of bacteria in the spleen and temporary infection in the liver in mice using *Bartonella birtlesii*. Accumulation of *Bartonella* in the spleen was attributed to the organ’s role in filtering and retention of infected erythrocytes rather than being an infective niche itself (67). It has been proposed that deformin, a deformation factor identified in some *Bartonella* species, causes physiological changes in infected erythrocyte membranes (68, 69), leading to the differential separation between infected and uninfected erythrocytes, with the damaged erythrocytes being cleared out by the spleen, as in the case of *Plasmodium falciparum* (70).

Haplotype analysis uncovered high genetic diversity and as expected, the highest diversity was attributed to isolates from *M. coucha,* which had the highest prevalence of *Bartonella* spp. in this study. The sampling locality with the highest genetic diversity was Groot Marico, in the North West province. Groot Marico is a small hamlet that relies greatly on agriculture, mining, and tourism (71). Natural habitats in this small town are often interspersed with farming land and human settlements. This spatial structuring due to habitat transformation often results in increased human-livestock-wildlife interfaces, which along with the high density of commensal and wild small mammals, presents an increased risk of zoonotic disease spread (72). The results of this study showed an extremely high probability of potential zoonotic *Bartonella* infection from small mammals.

Analysis of the median-joining and TCS networks showed the presence of diverse haplotypes among the sequences identified in this study. Propagation of bacteria in the digestive tract of the cat flea, lice, and ticks may allow interactions between different genotypes leading to gene recombination (73), which is one of the factors driving haplotype diversity (60). Gene recombination for *Bartonella* species and lateral gene transfer has been reported and is regarded as an evolutionary strategy within the genus responsible for pathogenicity (74). However, caution should be taken with our results, as haplotype diversity is sensitive to sample size (72). The haplotype diversity per site (π) should also be considered as it is insensitive to sample size.

From this study, we observed the existence of divergent *Bartonella* lineages in *Aethomys*, *Gerbillurus,* and *Mastomys* species supported by phylogenetic and haplotype network analyses. Bai et al. (75) also reported genetic heterogeneity of *Bartonella* spp. isolates in *Rattus* species. The median-joining network analysis showed that small mammals from the same geographic location or the same species harbored different *Bartonella* spp. genotypes. Possible interactions between the different genotypes could be a key component in the maintenance of *Bartonella* diversity (76).

To the best of our knowledge, this is the first study to assess nucleotide polymorphism variation of *Bartonella* spp. detected in small mammals covering an extensive geographic area in South Africa. However, the interpretation of diversity in this study remains limited by the smaller sequence coverage of the gene loci used compared to the size of the *Bartonella* genome. Therefore, whole-genome data would provide more comprehensive insight into the genetic variation observed in this study and the public health implications (77).

This study investigated the distribution of *Bartonella* spp. isolates in 183 wild small mammals collected from various parts of South Africa over a span of 9 years (2010–2018). The results from this study provide useful primary background data on the distribution and genetic diversity of *Bartonella* spp. isolates in wild small mammals across South Africa over the past 9 years. However, the major limitation of the study is that the number of animals screened over such a wide span of time is relatively small. Future research screening a larger sample size would provide more insight into the changes in the current spatial distribution and genetic diversity of *Bartonella* spp. in the country.

Another major limitation of this study was the low sensitivity of the *Bartonella gltA* and 16S-23S rRNA ITS PCR assays, compared to the *Bartonella rpoB* assay. The cyclic nature and low-level bacteremia, characteristic of *Bartonella* spp. infections make consistent diagnosis challenging. In a study by Drummond et al. (78), the use of a combination of different PCR tests as follows: (a) conventional PCR for two gene regions, the ITS and *gltA*; (b) nested PCR for the *ftsZ* gene; and (c) qualitative real-time PCR for the *gltA* gene increased the *Bartonella* spp. detection from 3.2% to 20.4%. However, this combination of tests still did not completely eliminate false negatives as some of the tested samples that had initially tested positive did not test positive again. Stochastic variation of PCR amplification is common when testing samples with low DNA copies (78). We therefore recommend the use of a combination of different PCR tests and different organ tissue for the accurate detection of *Bartonella* spp. in small mammals.

### Conclusions

The study confirmed the presence of a diverse collection of *Bartonella* lineages in small mammals in South Africa. A level of host specificity was observed, with clades comprising *Bartonella* spp. isolates from specific small mammal genera only. Among the sampled small mammal species, the highest *Bartonella* spp. occurrence was observed in *M. coucha*, *A. ineptus,* and *M. namaquensis. Mastomys coucha* displayed the highest infection rate, and this points to its suitability as a sentinel species in *Bartonella* surveillance programs. The high prevalence observed in abundant commensal rodent species *M. coucha*, *A. ineptus,* and *M. namaquensis*, together with the high levels of genetic diversity observed, calls for additional investigation of *Bartonella* prevalence, diversity, and zoonotic potential in the diverse small mammal biomes in South Africa and the importance of establishing a surveillance system of small mammal-associated *Bartonella* spp. with zoonotic importance in South Africa.

## Data Availability

All relevant data are available within the article and supplemental material. Representative *Bartonella* spp. sequences obtained from this study were deposited into the NCBI GenBank database, under accession numbers OR779532–OR779559.

## SUPPLEMENTAL MATERIAL

Fig. S1 aem.00842-24-s0001.tiff Phylogenetic tree inferred by maximum likelihood (ML) based on a 2,964-bp alignment of concatenated *Bartonella* 16S-23S rRNA ITS region, *gltA*, and *rpoB* genes. Numbers at nodes represent ML bootstrap support values with 1,000 repetitions and Bayesian posterior probabilities (BPP) greater than 70% (ML/BPP). Sequences from this study are shown in bold.

Tables S1 to S5 aem.00842-24-s0002.docx Haplotypes and BLAST results.

ASM does not own the copyrights to Supplemental Material that may be linked to, or accessed through, an article. The authors have granted ASM a non-exclusive, world-wide license to publish the Supplemental Material files. Please contact the corresponding author directly for reuse.
