# Supplementary figures and images for: Prevalence and genetic diversity of Bartonella spp. in wild small mammals from South Africa
Source: Appl Environ Microbiol. 2024 Jul 26;90(8):e00842-24. doi: 10.1128/aem.00842-24 (PMC11338311; doi:10.1128/aem.00842-24)

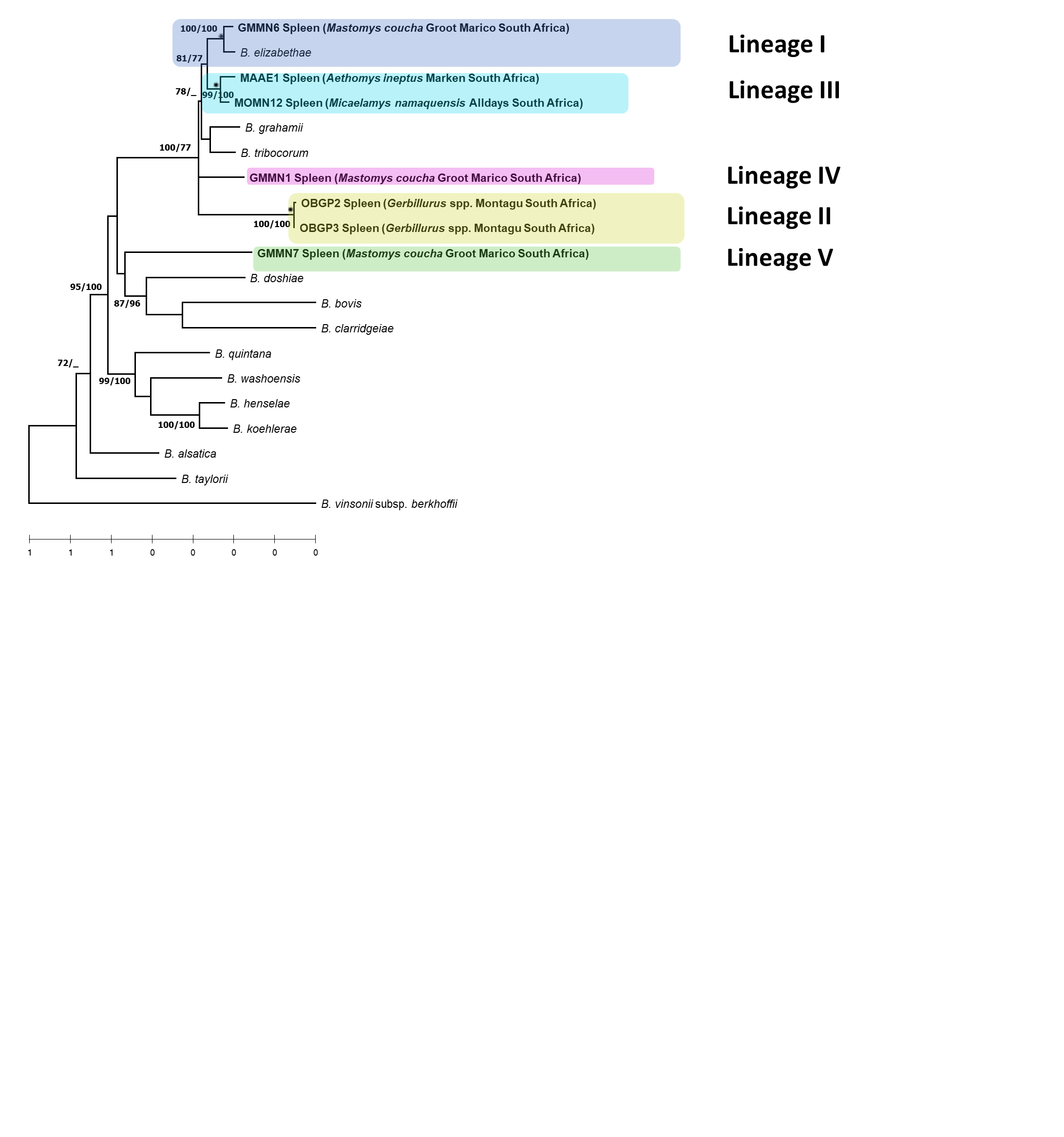

Supplement: Fig. S1 — Phylogenetic tree inferred by maximum likelihood (ML) based on a 2,964-bp alignment of concatenated Bartonella 16S-23S rRNA ITS region, gltA, and rpoB genes. Numbers at nodes represent ML bootstrap support values with 1,000 repetitions and Bayesian posterior probabilities (BPP) greater than 70% (ML/BPP). Sequences from this study are shown in bold. [file aem.00842-24-s0001.tiff]
