# Supplementary material for: Prevalence and genetic diversity of Bartonella spp. in wild small mammals from South Africa
Source: Appl Environ Microbiol. 2024 Jul 26;90(8):e00842-24. doi: 10.1128/aem.00842-24 (PMC11338311; doi:10.1128/aem.00842-24)
Supplement: Tables S1 to S5 — Haplotypes and BLAST results. [file aem.00842-24-s0002.docx]

**Table S1** Haplotypes detected from sequences in this study based on the Bartonella rpoB sequences.

| **Haplotype** | **Number of sequences** | **Samples** | **Small mammal species** |
| --- | --- | --- | --- |
| Hap_1 | 13 | DKAE24 Spleen, DKAE24 Lung, DKAE24 Liver, GMMN4 Spleen, GMMN4 Kidney, GMMN4 H&L, GMMN13 HL, GMMN13 Liver, GMMN13 Spleen, GMMN1 H&L, GMMN1 Spleen, GMMN16 H&L, GMMN1 H&L | *Mastomys coucha* |
| Hap_2 | 2 | GMMN6 Spleen,  GMMN6 Liver | *Mastomys coucha* |
| Hap_3 | 2 | GMMN7 Spleen  GMMN7 H&L | *Mastomys coucha* |
| Hap_4 | 10 | GMMN10 Spleen, GMMN10 Liver, GMMN10 Liver2, KNP12 Spleen, MAAE11 Spleen, MAAE13 Spleen, MOMN12 Lung, MOMN12 Spleen, MAAE1 Spleen, RPMN24 Liver | *Mastomys coucha,*  *Aethomys ineptus*  *Micaelamys namaquensis* |
| Hap_5 | 1 | MFGP 1 Spleen | *Gerbillurus* spp. |
| Hap_6 | 3 | MOMN6 Liver, MOMN6 Spleen,  MOMN6 Lung | *Aethomys chrysophilus* |
| Hap_7 | 2 | MOMN7 Spleen,  MOMN7 Liver | *Gerbillurus leucogaster* |
| Hap_8 | 3 | OBGP2 Spleen, OBGP2 Liver,  OBGP3 Spleen | *Gerbillurus* spp. |
| Hap_9 | 1 | OBRP9 Spleen | *Rhabdomys pumilio* |

**Table S2** Haplotypes detected from sequences in this study based on the Bartonella gltA sequences.

| **Haplotype** | **Number of sequences** | **Samples** | **Small mammal species** |
| --- | --- | --- | --- |
| Hap_1 | 5 | OBGP3Spleen, MFGP1Spleen, OBGP2Spleen, OBGP2Liver,  OBGP3Liver | *Gerbillurus* sp. |
| Hap_2 | 2 | GMMN1Spleen, GMMN4Spleen | *Mastomys coucha* |
| Hap_3 | 1 | GMMN6Spleen | *Mastomys coucha* |
| Hap_4 | 1 | GMMN7Spleen | *Mastomys coucha* |
| Hap_5 | 5 | GMMN10Spleen, MAAE1Spleen  MOMN6Spleen, MOMN12Lung, RPMN24Spleen | *Mastomys coucha, Aethomys chrysophilus, Micaelamys namaquensis* |

**Table S3** Haplotypes detected from sequences in this study based on the Bartonella 16S-23S rRNA ITS region sequences.

| **Haplotype** | **Number of sequences** | **Samples** | **Small mammal species** |
| --- | --- | --- | --- |
| Hap_1 | 1 | DKAE24 Spleen | *Mastomys coucha* |
| Hap_2 | 1 | GMMN1 Spleen | *Mastomys coucha* |
| Hap_3 | 1 | GMMN6 Spleen | *Mastomys coucha* |
| Hap_4 | 1 | GMMN7 Spleen | *Mastomys coucha* |
| Hap_5 | 1 | KNP12 Spleen | *Aethomys ineptus* |
| Hap_6 | 1 | MAAE1 Spleen | *Aethomys ineptus* |
| Hap_7 | 1 | MOMN12 Spleen | *Micaelamys namaquensis* |
| Hap_8 | 2 | OBGP2 Spleen, OBGP3 Spleen | *Gerbillurus* spp. |

**Table S4** Bartonella spp. gltA sequences from wild small mammals in South Africa and their closest BLASTn matches.

| **Sample ID** | **Closest GenBank match (%)** | **GenBank Accession number** | **Closest GenBank species match (%)** | **GenBank Accession number** | **Host species**  **Retrieved from** |
| --- | --- | --- | --- | --- | --- |
| OBGP3 *gltA* | *Bartonella* sp. (97.07) | JX428749.1 | *Bartonella gabonensis* strain 669 (96.67) | MT274297.1 | Savannah rodent *Lophuromys sikapusi* Gabon |
| GMMN1 *gltA* | *Bartonella* sp. AN-nh3 (100) | AJ583114.1  South Africa | *Bartonella grahamii* as4aup (96.42) | CP001562.1 | Mouse |
| GMMN4 *gltA* | *Bartonella* sp. AN-nh3 (100) | AJ583114.1  , wild small mammal South Africa | *Bartonella grahamii* as4aup (96.57) | CP001562.1 | Mouse |
| GMMN6 *gltA* | Uncultured *Bartonella* sp. clone Pd5700t (99.68) | FJ851115.1  Equatorial Guinea, wild small mammal | *Bartonella elizabethae* strain BR02 (99.67) | GU056192.1 | Ectoparasites of stray animals, Taiwan |
| GMMN7 *gltA* | Uncultured *Bartonella* sp. clone 1034-1 (98.97) | MF443365.1  Fleas from Uganda | *Bartonella vinsonii subsp. arupensis* isolate MP2 (92.53) | MK984790.1 | Prairie rodents, Illinois |
| GMMN10 *gltA* | Uncultured *Bartonella* sp. clone Pd5695t (97.38) | FJ851114.1  Equatorial Guinea, wild small mammal | *Bartonella tribocorum* strain B29906  (96.41) | KT327031.1 | Libyan jird (*Meriones libycus*) |
| MAAE1 *gltA* | Uncultured *Bartonella* sp. clone Pd5695t (96.46) | FJ851114.1  Equatorial Guinea, wild small mammal | *Bartonella tribocorum* strain B29906  (95.88) | KT327031.1 | Libyan jird (*Meriones libycus*) |
| **Sample ID** | **Closest GenBank match (%)** | **GenBank Accession number** | **Closest GenBank species match (%)** | **GenBank Accession number** | **Host species**  **Retrieved from** |
| MFGP1 *gltA* | *Candidatus Bartonella gerbillinarum* strain OE12C  MH618808.1  (87.72) | Wild gerbils, Israel | The same as highest match |  |  |
| MOMN6 *gltA* | Uncultured *Bartonella* sp. clone Pd5695t (99.09) | FJ851114.1  Equatorial Guinea, wild small mammal | *Bartonella tribocorum* strain B29906  (98.18) | KT327031.1 | Libyan jird (*Meriones libycus)* |
| MOMN12 Lung *gltA* | *Bartonella* sp. TT0105  (97.07) | FJ667572.1  Field rodents in Taiwan | *Bartonella tribocorum* strain B29906  (97.06) | KT327031.1 | Libyan jird (*Meriones libycus*) |
| OBGP2 *gltA* | *Bartonella* sp. Cg1ug  (97.07) | GenBank: JX428749.1  Invasive and indigenous rodents, Uganda | *Bartonella gabonensis* strain 669 (96.74) | MT274297.1 | Savannah rodent *Lophuromys sikapusi* Gabon |
| **Sample ID** | **Closest GenBank match (%)** | **GenBank Accession number** | **Closest GenBank species match (%)** | **GenBank Accession number** | **Host species**  **Retrieved from** |
| OBGP3 Liver *gltA* | *Bartonella* sp. Cg1ug  (96.70) | GenBank: JX428749.1  Invasive and indigenous rodents, Uganda | *Bartonella gabonensis* strain 669 (96.37) | MT274297.1 | Savannah rodent *Lophuromys sikapusi* Gabon |
| RPMN24 *gltA* | Uncultured *Bartonella* sp. clone Pd5695t (97.41) | FJ851114.1  Equatorial Guinea, wild small mammal | *Bartonella tribocorum* strain B29906  (97.05) | KT327031.1 | Libyan jird (*Meriones libycus*) |

**Table S5** Bartonella spp. 16S-23S rRNA ITS region sequences from wild small mammals in South Africa and their closest BLASTn matches.

| **Sample ID** | **Closest GenBank match (%)** | **GenBank Accession no. Country & Host** | **Closest GenBank species match (%)** | **GenBank Accession no. Country & Host** |
| --- | --- | --- | --- | --- |
| DKAE24_ITS | *B. japonica*  (87.3) | AB498007.2 | NA | NA |
| GMMN1_ITS | *Bartonella* sp. An2ug (99) | JX428753.1  Uganda, Invasive and Indigenous rodents | *Bartonella tribocorum* isolate TR-19  (91) | DQ480757.1,  USA, fleas from rodents |
| GMMN6_ITS | *Bartonella* sp. RN28BJ  (99) | EF213776.1  China,  *Rattus norvegicus* | *Bartonella mastomydis* strain 008  (96) | MN158196.1  Senegal,  *Mastomys erythroleucus* |
| GMMN7_ITS | *Bartonella* sp. An27ug  (100) | JX428757.1  Uganda,  *Arvicanthis niloticus* | *Bartonella florencae* strain R4  (93) | HM622140.1,  France,  *Crocidura russula* |
| **Sample ID** | **Closest GenBank match (%)** | **GenBank Accession no. Country & Host** | **Closest GenBank species match (%)** | **GenBank Accession no. Country & Host** |
| KNP12_ITS | *Bartonella elizabethae*  (88) | LR746177.1  France,  *Stenoponia tripectinata tripectinata* | *Bartonella elizabethae*  (88) | NA |
| MOMN12_ITS | *Candidatus Bartonella* sp. ML-2022b strain R18  (90) | OM459734.1  Senegal,  *Cricetomys gambianus* | *Bartonella elizabethae* (89) | L35103.1 |
| OBGP2 ITS | *Bartonella* sp. HT3-1  (87) | AB602566.1,  Egypt  *Jaculus jaculus* | *Bartonella pachyuromydis* (86%) | AB602561.1  Netherlands,  *Pachyuromys duprasi* |
| OBGP3 ITS | *Bartonella* sp. HT3-1  (89) | AB602566.1,  Egypt  *Jaculus jaculus* | Bartonella pachyuromydis (87) | AB602561.1  Netherlands,  *Pachyuromys duprasi* |
